# Optimization of silver nanoparticle synthesis by chemical reduction and evaluation of its antimicrobial and toxic activity

**DOI:** 10.1186/s40824-019-0173-y

**Published:** 2019-12-19

**Authors:** Catalina Quintero-Quiroz, Natalia Acevedo, Jenniffer Zapata-Giraldo, Luz E. Botero, Julián Quintero, Diana Zárate-Triviño, Jorge Saldarriaga, Vera Z. Pérez

**Affiliations:** 1 Centro de Bioingeniería, Grupo de investigaciones en Bioingeniería, Universidad Pontificia Bolivariana, circular 1 No. 73-76, Medellín, 050031 Colombia; 2 Grupo de Investigación de Biología de Sistemas,Universidad Pontificia Bolivariana, Cl 78B No. 72A-109, Medellín, 050031 Colombia; 3 Universidad de Antioquia, Cl.67 No. 53-108, Medellín, 050010 Colombia; 4 Laboratorio de Inmunología y Virología, Universidad Autónoma de Nuevo León, Ave. Pedro de Alba S/N Ciudad Universitaria San Nicolás de los Garza, Monterrey, 64450 México; 5 Grupo de Investigación Sobre Nuevos Materiales, Universidad Pontificia Bolivariana, Cq.1 No. 70-01, Medellín, 050031 Colombia; 6 Facultad de Ingeniería Eléctrica y Electrónica, Medellín, 050031 Colombia

**Keywords:** Silver nanoparticles, Design of experiments, Response surface methodology, Antimicrobial activity, Cytotoxicity

## Abstract

**Background:**

Chemical reduction has become an accessible and useful alternative to obtain silver nanoparticles (AgNPs). However, its toxicity capacity depends on multiple variables that generate differences in the ability to inhibit the growth of microorganisms. Thus, optimazing parameters for the synthesis of AgNPs can increase its antimicrobial capacity by improving its physical-chemical properties.

**Methods:**

In this study a Face Centered Central Composite Design (FCCCD) was carried out with four parameters: *AgNO*_3_ concentration, sodium citrate (TSC) concentration, *NaBH*_4_ concentration and the pH of the reaction with the objective of inhibit the growth of microorganisms. The response variables were the average size of AgNPs, the peak with the greatest intensity in the size distribution, the polydispersity of the nanoparticle size and the yield of the process. AgNPs obtained from the optimization were characterized physically and chemically. The antimicrobial activity of optimized AgNPs was evaluated against *Staphylococcus aureus*, *Escherichia coli*, *Escherichia coli* AmpC resistant, and *Candida albicans* and compared with AgNPs before optimization. In addition, the cytotoxicity of the optimized AgNPs was evaluated by the colorimetric assay MTT (3- (4,5- Dimethylthiazol- 2- yl)- 2, 5 - Diphenyltetrazolium Bromide).

**Results:**

It was found that the four factors studied were significant for the response variables, and a significant model (p < 0.05) was obtained for each variable. The optimal conditions were 8 for pH and 0.01 M, 0.0 6M, 0.01 M for the concentration of TSC, *AgNO*_3_, and *NaBH*_4_, respectively. Optimized AgNPs spherical and hemispherical were obtained, and 67.66% of it had a diameter less than 10.30 nm. A minimum bactericidal concentration (MBC) and minimum fungicidal Concentration (MFC) of optimized AgNPs was found against *Staphylococcus aureus*, *Escherichia coli*, *Escherichia coli* AmpC resistant, and *Candida albicans* at 19.89, 9.94, 9.94, 2.08 *μ*g/mL, respectively. Furthermore, the lethal concentration 50 (*LC*_50_) of optimized AgNPs was found on 19.11 *μ*g/mL and 19.60 *μ*g/mL to Vero and NiH3T3 cells, respectively.

**Conclusions:**

It was found that the factors studied were significant for the variable responses and the optimization process used was effective to improve the antimicrobial activity of the AgNPs.

## Background

Silver ions have been known for their effectiveness against a wide range of microorganisms [1]. The antimicrobial activity of silver nanoparticles (AgNPs) has been confirmed in both Gram-positive and Gram-negative bacteria as well as in fungus [2, 3]. AgNPs have been used in several medical application such as sunscreen lotions, burn treatment, wound dressings, textiles, dental materials, bone implants and medical device coating among others [4–6].

The antimicrobial effect of AgNPs relies on physic-chemical characteristics like size, shape, distribution and concentration [5]. The mechanism of action has been associated to several factors including damage to the cell membrane of bacteria or the plasma membrane of fungi that causes the loss of cellular components [5, 7, 8], disruption of the respiratory chain and synthesis of adenosine triphosphate (ATP), which affects the cellular energy source causing death of the microorganism, damage to deoxyribonucleic acid (DNA) and disruption of cell replication [4, 7, 8]. However, it is expected that AgNPs does not cause cellular damage or affect beneficial microorganisms [2]. As a result, the cytotoxic activity is important to define their applications [1].

Methods of synthesis for AgNPs have gained a lot of attention recently due to the need to find more efficient ways to obtain nanoparticles. The bottom-up chemical technique is one of the most use methods in terms of nanoparticle production. This method is low cost and has a large-scale production capacity. It is based on the reduction of a metal salt via a reducing agent in the presence of a protective material. AgNPs formation begins with generating a neutral silver atom that forms *Ag*^2+^ precursor. Subsequently, more atoms are added and this forms a cluster that allows to control shape and size of the nanoparticles [1, 5].

Due to the advances in AgNPs production with different characteristics, and because of physico-chemical properties effects on microorganisms, characterization techniques have been developed. Those techniques allow analyzing AgNPs structure, morphology, composition, and behavior using technologies such as visible ultraviolet spectroscopy (UV -Vis), dynamic light scattering (DLS), and transmission electron microscopy (TEM) [5]. UV-Vis evaluates surface plasmon resonance (LSPR) of metal nanoparticles, provides information on its size and has been used as a benchmark for the performance of the nanoparticle synthesis process [9, 10]. On the other hand, DLS uses a monochromatic light source to measure the size, structure, and distribution of nanomaterials [11, 12]. The electrical potential to measure the electrostatic attraction or repulsion capacity between particles can be measured through the evaluation of the zeta potential by DLS [11, 12]. In addition, it is possible to obtain nanoparticle images with a resolution of up to 0.1 nm employing TEM [13].

There are several studies that optimize the synthesis parameters of AgNPs, mainly to reduce the size and improve their physico-chemical properties [14, 15]. However, even if there are well-established techniques for the preparation of metallic nanoparticles, it is necessary to investigate simple synthesis methods, which require short reaction times and low cost to obtain nanoparticles with greater antimicrobial activity [16].

In this study, a Face Centered Central Composite Design (FCCCD) was carried out to optimize the synthesis of AgNPs obtained based on the method of chemical reduction [17]. These nanoparticles were established as the reference AgNPs, and their antimicrobial activity was evaluated. The design was performed with four parameters: *AgNO*_3_ concentration, sodium citrate (TSC) concentration, *NaBH*_4_ concentration, and the pH of the reaction to obtain a better antibacterial effect of the reference AgNPs. The optimized AgNPs were characterized by evaluating some of their physical-chemical properties and antimicrobial activity. Additionally, the cytotoxic effect was assessed using NiH3T3 and Vero cell lines. NiH3T3 is the standardized cell line recommended by the Organization for Economic Cooperation and Development (OECD) as the in vitro model to test the cytotoxicity of manufactured nanomaterials [18]. The Vero cell line was used to determinate the cytotoxicity of the AgNPs over a blood filter cell, like the kidneys, since it has been determined that the hemocompatibility of nanoparticles is a prior requirement for its use in medical products [19].

## Materials and methods

**Fig. 1:**
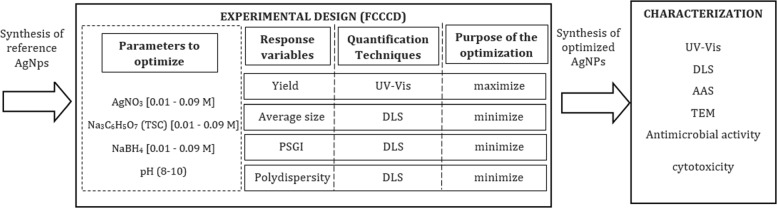
Optimization process for the synthesis of silver nanoparticles using experimental design

A schematic diagram of the optimization process is depicted in Fig. 1.

### Synthesis of silver nanoparticles

Briefly, 5 mL of sodium citrate 0.05 M (TSC, Sigma-Aldrich CAS 6132-04-3) and 5 mL of silver nitrate 0.05 M (*AgNO*_3_, PANREAC CAS 7761-88-8) were added to 185 mL of water type 1 (Milli Q ^*Ⓡ*^) in a cold bath between 6 ^∘^C to 10 ^∘^C. The solution was stirred for 3 min at 3000 RPM. Subsequently, 5 mL of sodium borohydride 0.05 M (*NaBH*_4_, Sigma-Aldrich CAS 16940-66-2) was dripped slowly. The pH was adjusted to 10 with sodium hydroxide 1.25 M (NaOH, PANREAC CAS 1310-73-2). The nanoparticles obtained were stored in amber bottles at 4 ^∘^C. These nanoparticles were the reference AgNPs (Ref-AgNPs) for the study.

### Experimental design and optimization

An optimization process of Ref-AgNPs was carried out, with the purpose of improving some of its physico-chemical properties. A FCCCD was executed using the Design Expert Version 7.0.0 software (Stat-Ease, USA) with four parameters: *AgNO*_3_ concentration (0.01 - 0.09 M), TSC concentration (0.01 - 0.09 M), *NaBH*_4_ concentration (0.01 - 0.09 M) and the pH of the reaction (8 – 10). The response variables were established according to the synthesis performance. The variables were, i) area under the curve of the UV-Vis absorbance spectrum; ii) average size of AgNPs; iii) the greatest intensity peak in the size distribution of AgNPs (PSGI); iv) the polydispersity of AgNPs. These dependent variables were quantified with the following techniques:

#### Ultraviolet- visible spectroscopy (UV-Vis)

This technique was used to determine the plasmonic surface resonance. A spectrophotometer UV probe 1601pc Shimadzu was used for reading the absorbance between 350 – 420 nm. The yield of the synthesis was estimated as the area under the curve of the ultraviolet absorbance of the nanoparticles evaluated [21].

#### Dynamic light scattering (DLS)

DLS was used to establish the average size of AgNPs, PSGI and the polydispersity. A Zetasizer Nano Series Malvern Instruments (USA) was used. The samples were diluted in water type 1 at controlled temperature (23 ^∘^C) to obtain a dilution factor that allow a reliable reading. Three measurements were made, each with 30 s of balance and 15 runs of 10 s of duration [22].

The optimization process sought to maximize the yield with an importance of 5. On the other hand, it was work towards minimize the average size, the peak size with greater intensity, and the polydispersity related to the size of AgNPs, with importance of 3, 4 and 5, respectively. The least squares multiple regression method was used.

The experimental data was adjusted using the second order polynomial equation by comparing the coefficient of determination (*R*^2^) and the adjusted coefficient of determination (*R*^2^−*adj*). The analysis of variance (ANOVA) was used to evaluate the statistical significance of the independent variables from the obtained models (with a confidence level of 95%). The accuracy of the optimal conditions was evaluated by calculating the relative and absolute errors between the responses predicted by the model and those obtained experimentally under optimal conditions.

### Characterization of AgNPs

#### Physico-chemical characterizations of optimized AgNPs

Optimized AgNPs (Opt-AgNPs) were characterized physico-chemically by the UV-Vis and DLS methods as described in the design of the experiment. In addition, AgNPs were characterized by atomic absorption (AAS), zeta potential, and transmission electron microscopy (TEM) as described follows:

*Atomic absorption spectroscopy (AAS)*

The silver concentration in each synthesis was determined with the flame method using the AAS technique using a Thermo Scentific ICE 3000, USA [23]. A sample of the colloidal solution of the undiluted nanoparticles was nebulized and disseminated as an aerosol to measure the parts per million Ag.

*Zeta potential*

The Zeta potential was determined by Laser Doppler Electrophoresis using a Zetasizer Nano ZS and the Zetasizer software. The nanoparticles were diluted in water type 1 at controlled temperature (23 ^∘^C) and three measurements were made, each of them with 30 s of equilibrium and 15 runs of 10 s in length [24].

*Transmission electron microscopy (TEM)*

The size and morphology of the samples were confirmed by transmission electron microscopy (TEM) using a Tecnai F20 Super Twin TMP, FEI. The samples were prepared using a drop of approximately 60 nm thickness of each suspension and deposited on a carbon membrane [21, 25].

#### Evaluation of the antimicrobial effect of AgNPs

The antibacterial and antifungal activity of Ref-AgNPs and Opt-AgNPs was evaluated with the macrodilution and microdilution techniques [21, 26]. The microorganism used where *Staphylococcus aureus* ATCC 25923, *Escherichia coli* ATCC 25922, *Escherichia coli* AmpC resistant, and *Candida albicans* ATCC 14053. The minimum bactericidal concentration (MBC) and the minimum fungicidal concentration (MFC) was evaluated to establish the bactericidal and antifungal capacity of the AgNPs, respectively.

Briefly, each bacterium species were seeded on Müller Hinton agar (BD, REF 211438) and incubated for 24 h at 37 ^∘^C. Subsequently, a sample of each microorganism was cultured between 12 and 24 h in Brain Heart Infusion liquid medium (BHI, BD REF 211065) at 37 ^∘^C in order to reach log phase. Each bacterium was adjusted to 5 ×10^4^ CFU/mL using a spectrophotometer (Genesys 20, Thermo Scientific USA). The microorganism were diluted at different concentrations of AgNPs (2.48, 4.97, 9.94, 19.89, 29.83 and 39.78 *μ*g/mL) each one with 2.5 ×10^4^ UCF/mL bacteria. 150 *μ*l at 0.02M TSC was added to each nanoparticle solution. Each dilution was incubated for 24 h at 37 ^∘^C in a shaking incubator (Rosy 1000, Thermolyne USA), under constant stirring at 75 RPM. A volume of 10 *μ*L of these dilutions were seeded on Müeller-Hinton agar and incubated at 37 ^∘^C for 24 h. The MBCs were determined visually as the lowest concentration of AgNPs that visually inhibits 99.9% growth of microorganisms [26]. For each assay, there were a number of controls such as microorganism growth, AgNPs, diluent and TSC sterility controls.

On the other hand, the MFC of AgNPs on *Candida albicans* ATCC 14053 was evaluated through the microdilution technique in broth [26]. The fungus was seeded on Sabouraud agar (BD, REF 210950) for 48 h at 37 ^∘^C. The microorganism was subculture for 48 h in BHI liquid medium at 37 ^∘^C. Apart from that, AgNPs concentrations were obtained from 0.12 to 3.97 *μ*g/mL by diluting with water type 1 (Milli Q ^*Ⓡ*^). The fungus suspension was adjusted to 2.5 ×10^3^ CFU/mL using a Genesys 20 spectrophotometer (Thermo Scientific USA). On a 96 well flat bottom microplate (Costar REF 3599), 20 *μ*L of each dilution of AgNPs, 220 *μ*L of BHI culture medium, and 10 *μ*L of the microorganism were added. Sterility and sensitivity controls of the microorganism were cultured using fluconazole 99% (Pfizer, lot 04821) at 10 and 5 *μ*g/mL, and viability control. In each well, a final volume of 250 *μ*L was obtained.The microplate was incubated at 37 ^∘^C and kept under agitation at 60 RPM for 24 h in an incubator-agitator (Rosy 1000, Thermolyne USA). After 24 h, the absorbance of each well was read at a wavelength of 530 nm and 10 *μ*L of each well was seeded in Petri dishes with Sabouraud agar. The dishes were incubated for 48 h at 37 ^∘^C. The antibacterial and antifungal activity was evaluated in triplicate to obtain the median CFU/mL. The MFC for the fungus of AgNPs was evaluated visually and using the Probit regression method and the IBM SPSS Statistics 24.0 software was used for statistical analysis.

#### Evaluation of the cytotoxic effect of optimized AgNPs

The evaluation of the cytotoxic effect of Ref-AgNPs was carried out on NiH3T3 and Vero cells through a MTT assay ((3-[4,5-dimethylthiazol-2-yl]-diphenyl tetrazolium bromide)) of cellular viability. The cells were cultured as monolayer in Dulbecco’s Modified Eagle Medium (DMEM) supplemented with 5% of fetal bovine serum (SFB) and 1% antibiotics (penicillin and streptomycin). The cultures were maintained in incubator at 37 ^∘^C with 5% *CO*_2_ atmosphere in a 25 cm^2^ culture flask. Cells were trypsinized (0,05% trypsin EDTA) and seeded in 96-well plates, for cytotoxicity assay. 5 ×10^3^ cells in 200 *μ*l of medium were seeded in each well. The plates were incubated at 37 ^∘^C with 5% *CO*_2_ atmosphere for 24 h to allow the cellular adherence. Then, the medium was removed and new medium was added with AgNPs at different concentrations (0, 5, 10, 15, 20, 40, 60, 70, 80, 90 *μ*g/mL) and in different wells. The cells were incubated for 24 h at 37 ^∘^C with 5% *CO*_2_ atmosphere. After that, the medium with AgNPs was removed and was added 100 *μ*l medium with 10 *μ*l of MTT in each well and the plates were incubated for 2 h at 37 ^∘^C. Subsequently, 100 *μ*l of Dimethylsulfoxide (DMSO) was added to each well and its absorbance was measured using a microplate reader (Synergy HT Biotek ^*Ⓡ*^) at 570 nm [27]. Untreated cells with AgNPs was used as control. MTT assay was performed with six replicas for the different AgNPs concentrations and to each cellular line. The data was statistically analyzed with IBM SPSS Statistics software to determine the cellular viability and recognize if AgNPs cause cytotoxic effect. Lethal concentration 20 (*LC*_20_) and lethal concentration 50 (*LC*_50_) of AgNPs optimized were obtained by Probit analysis that assesses the mortality percentage for each AgNPs concentration evaluated. Percentage of cellular viability was calculated taking the cellular control as 100% of viability [28].

## Results

### Experimental design

A central composite design was obtained using the Response Surface Methodology (RSM) with the objective of identifying the interactions between the parameters such as *AgNO*_3_ concentration (0.01 - 0.09 M), TSC concentration (0.01 - 0.09 M), *NaBH*_4_ concentration (0.01 - 0.09 M) and the pH of the reaction (8 - 10). The objective was to increase the yield in the production of AgNPs, as well as to reduce the average size and the polydispersity in the size of the nanoparticles.

The Design of Experiments (DOE) showed 29 experimental runs which are presented in a randomized manner in Table 1. The highest response for the UV-Vis area was 15.00 which was obtained with TSC [0.09 M], *AgNO*_3_ [0.09 M], *NaBH*_4_ [0.09 M] and pH 8. On the other hand, the average size of the lowest nanoparticle, the peak of the highest intensity of the smallest size and the lowest polydispersity were 12.39, 15.17 and 0.112, which were obtained with *AgNO*_3_ at a concentration of 0.01 M and pH 8. The seven tests with area 0.00 of the UV-Vis spectrum, were synthesized with pH 12. They had the largest average sizes of AgNPs as well as the most significant peaks in size with the highest intensity. Besides, among these essays were found the 3 with greater polydispersity in size.

**Table 1:** Three-variable FCCCD design with four responses for the synthesis of AgNPs

Run	TSC(M)	*AgNO*_3_	*NaBH*_4_(M)	pH	Yield (UV-Vis area)	Size (nm)	PSGI(nm)	Polydispersity
1	0.05	0.05	0.01	10	7.98 ± 0.72	70.81 ± 8.90	55.61 ± 15.41	0.15
2	0.09	0.09	0.09	8	15.00 ± 1.37	17.66 ± 7.77	65.72 ± 13.30	0.64
3	0.05	0.05	0.05	8	8.55 ± 0.78	21.18 ± 7.35	99.40 ± 71.08	0.81
4	0.09	0.01	0.01	8	0.85 ± 0.08	159.60 ± 6.59	15.17 ± 4.78	0.31
5	0.05	0.05	0.05	12	7.87 ± 0.72	280.90 ± 27.47	461.60 ± 91.00	0.72
6	0.05	0.05	0.05	10	7.63 ± 0.69	13.66 ± 1.33	52.30 ± 6.72	0.55
7	0.05	0.05	0.09	10	8.10 ± 0.74	33.14 ± 3.24	99.99 ± 7.40	0.56
8	0.09	0.09	0.09	12	0.00 ± 0.00	8346.00 ± 816.00	8346.00 ± 816.00	1.00
9	0.05	0.05	0.05	10	7.97 ± 0.73	15.45 ± 1.51	66.73 ± 4.47	0.65
10	0.05	0.01	0.05	10	1.62 ± 0.15	372.60 ± 2.53	23.13 ± 4.08	0.55
11	0.01	0.09	0.01	8	2.83 ± 0.25	53.56 ± 9.14	46.28 ± 6.39	0.17
12	0.09	0.01	0.01	12	0.00 ± 0.00	3724.00 ± 364.20	3724.00 ± 364.20	1.00
13	0.09	0.05	0.05	10	7.82 ± 0.72	19.61 ± 1.91	87.18 ± 60.20	0.71
14	0.09	0.09	0.01	12	0.00 ± 0.00	3181.00 ± 311.10	3181.00 ± 311.10	1.00
15	0.05	0.05	0.05	10	8.02 ± 0.73	20.88 ± 2.04	96.12 ± 59.81	0.80
16	0.01	0.09	0.09	12	0.00 ± 0.00	37900.00 ± 3706.62	37900.00 ± 3706.62	0.23
17	0.05	0.05	0.05	10	7.76 ± 0.71	13.65 ± 1.33	59.91 ± 39.54	0.56
18	0.01	0.01	0.09	12	0.00 ± 0.00	30200.00 ± 2953.56	30200.00 ± 2953.56	0.28
19	0.09	0.01	0.09	8	1.37 ± 1.12	12.39 ± 1.21	36.56 ± 16.35	0.55
20	0.05	0.09	0.05	10	14.05 ± 1.28	15.96 ± 1.56	68.58 ± 48.06	0.62
21	0.01	0.01	0.09	8	1.13 ± 0.10	19.04 ± 1.86	68.14 ± 37.47	0.79
22	0.05	0.05	0.05	10	7.79 ± 0.71	26.95 ± 2.63	114.40 ± 76.30	0.89
23	0.09	0.01	0.09	12	0.94 ± 0.08	945.60 ± 92.47	741.50 ± 151.60	0.65
24	0.01	0.09	0.01	12	0.00 ± 0.00	985.80 ± 96.41	985.80 ± 18.03	0.90
25	0.09	0.09	0.01	8	3.29 ± 0.29	57.50 ± 5.62	125.00 ± 108.70	0.52
26	0.01	0.01	0.01	8	1.40 ± 0.13	40.87 ± 3.99	36.52 ± 15.27	0.11
27	0.01	0.05	0.05	10	7.42 ± 0.68	49.17 ± 4.80	101.6 ± 72.71	0.52
28	0.01	0.09	0.09	8	4.41 ± 0.40	45.59 ± 4.45	98.73 ± 65.20	0.51
29	0.01	0.01	0.01	12	0.02 ± 0.00	15.25 ± 1.49	62.07 ± 48.51	0.62

Values are expressed as mean ± standard deviation (*n* = 3). PSGI: peak size with greater intensity

### Validation of the experimental model using residuals

**Fig. 2:**
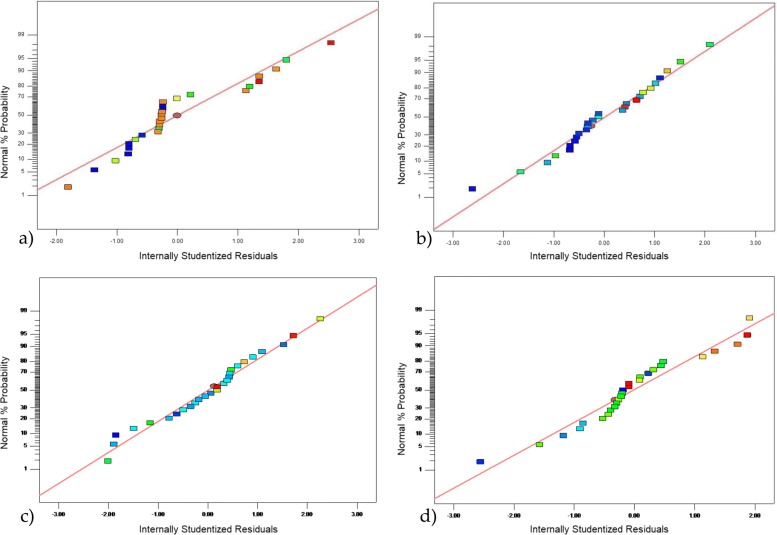
Normal plot of residuals; **a**) for the performance in the production of AgNPs; **b**) for the average size of AgNPs; **c**) for the highest intensity peak of AgNPs; **d**) for polydispersity of the size of AgNPs

**Fig. 3:**
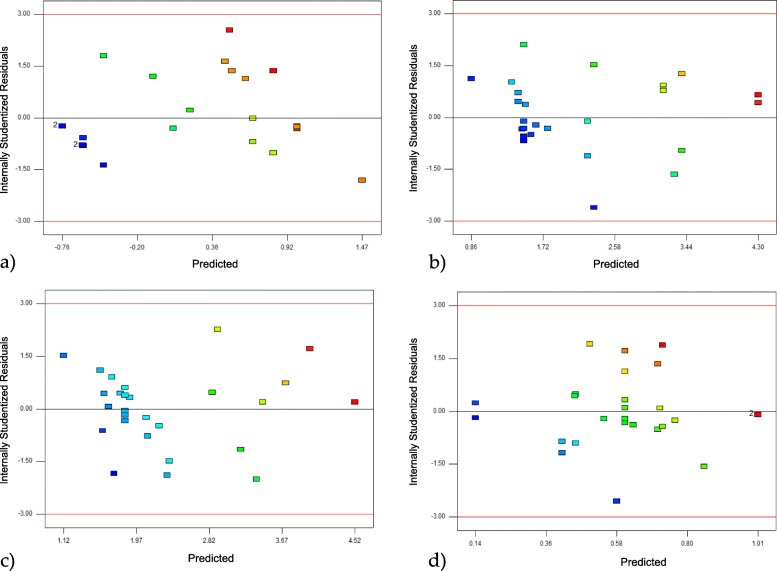
Graph of residuals vs. predicted model; **a**) for the performance in the production of AgNPs; **b**) for the average size of AgNPs; **c**) for the highest intensity peak of AgNPs; **d**) for polydispersity of the size of AgNPs

**Fig. 4:**
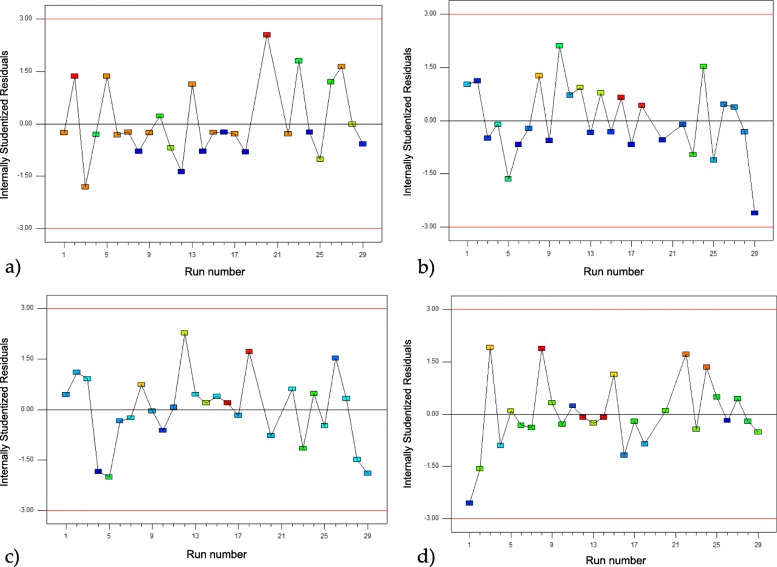
Residual graph vs. observation order; **a**) for the performance in the production of AgNPs; **b**) for the average size of AgNPs; **c**) for the highest intensity peak of AgNPs; **d**) for polydispersity of the size of AgNPs

The optimal conditions and validation of the design of the experiment were determined using Design Expert Software. It was based on the analysis of the normal residual graphs for each of the response variables (Fig. 2), the analysis of the residual vs. predicted graphs of the validation model (Fig. 3), and the residual vs. observation order graphs (Fig. 4). It was observed that the experimental design presented a linear relationship in the distribution of errors. The assumption of normality was verified by the normal probability graph, the independence between residues, and the normal and random distribution between positive and negative residues.

On the other hand, the validation of optimal values prediction was assessed with the calculation of the relative and absolute errors was accomplished between the responses predicted by the model versus the ones obtained experimentally under optimal conditions (Table 3).

### Statistical analysis of experiments

ANOVA analysis were performed in each experimental unit, where the quadratic model was found to be significant (P < 0.05). Table 2 shows ANOVA results and the statistical description for the model obtained in each of the responses.

**Table 2:** ANOVA and statistical description by FCCCD

	*p* value			
Source	Yield	Size	PSGI	Polydispersity
Model	<0.000	<0.000	<0.000	0.000
A-TSC	0.359	0.882	0.572	0.001
B- *AgNO*_3_	0.093	—	0.016	—
C- *NaBH*_4_	—	0.314	0.023	0.577
D-pH	<0.000	<0.000	<0.000	0.025
AC	—	0.007	0.002	—
BD	0.020	—	—	—
CD	—	0.014	0.037	0.001
*A*^2^	0.016	—	—	—
*B*^2^	0.001	—	—	—
*D*^2^	—	0.000	0.000	—
Lack of fit	<0.000	0.004	0.026	0.401
R2	0.856	0.802	0.878	0.612
Adjusted R2	0.814	0.743	0.834	0.541

The relationships between the dependent variables (yield, size, PSGI, polydispersity) and the independent variables (TSC, *AgNO*_3_, *NaBH*_4_, and pH) are expressed by the following regression equations:

The yield of AgNPs synthesis (yield) model is given below in Eq. 1.

1 $$ \begin{aligned} \log_{10}(yield + 0.15) = &-7.26071 x 10^{-3} + 30.61911 \times TSC \\ &+ 72.43530 \times {AgNO}_{3} \\ - 0.10162 \times pH &- 2.81328 \times {AgNO}_{3} \times pH - 287.25097 \\ &\times TSC^{2} - 406.12621 \times {{AgNO}_{3}}^{2} \end{aligned}  $$

The average size of AgNPs (size) model is given below in Eq. 2.

2 $$ \begin{aligned} \log_{10}(size)= 22.59344 &+ 13.17486 \times TSC - 33.27239 \times {NaBH}_{4}\\ &- 4.56699 \times pH \\ - 273.76717 \times TSC &\times {NaBH}_{4} + 5.05783 \times {NaBH}_{4} \times pH\\ &+ 0.23722 \times pH^{2} \end{aligned}  $$

The peak size with greater intensity of AgNPs (PSGI) model is given below in Eq. 3.

3 $$ \begin{aligned} \log_{10}(PSGI) =& 15.22917 + 9.55131 \times TSC + 6.56465\\ &\times {AgNO}_{3} - 12.83922 \\ * {NaBH}_{4} - 3.17281 &\times pH - 218.23765 \times TSC \times {NaBH}_{4} + 2.98745 \\ &\times {NaBH}_{4} \times pH + 0.17228 \times pH^{2} \end{aligned}  $$

The AgNPs size polydispersity (Polydispersity) model is given below in Eq. 4.

4 $$ \begin{aligned} Polydispersity = -1.24875 &+ 3.87813 \times TSC + 22.27832\\ &\times {NaBH}_{4} +0.16253 \\ &\times pH -2.16396 \times {NaBH}_{4} \times pH \end{aligned}  $$

All the models obtained a significant value (p < 0.05), where the pH was statistically significant. The coefficient of determination (*R*^2^) indicates the correlation between experimental and predicted data. In addition, adjusted *R*^2^ and *R*^2^ corroborate the significance of the models. According to the coefficients of each effect analyzed, the concentration of *AgNO*_3_ has greater effect on the yield in the production of AgNPs, while TSC has greater effect on the size and maximum peak of the size of the nanoparticles. In terms of polydispersity in the size of AgNPs, the concentration of *NaBH*_4_ is the factor with the greatest effect.

**Fig. 5:**
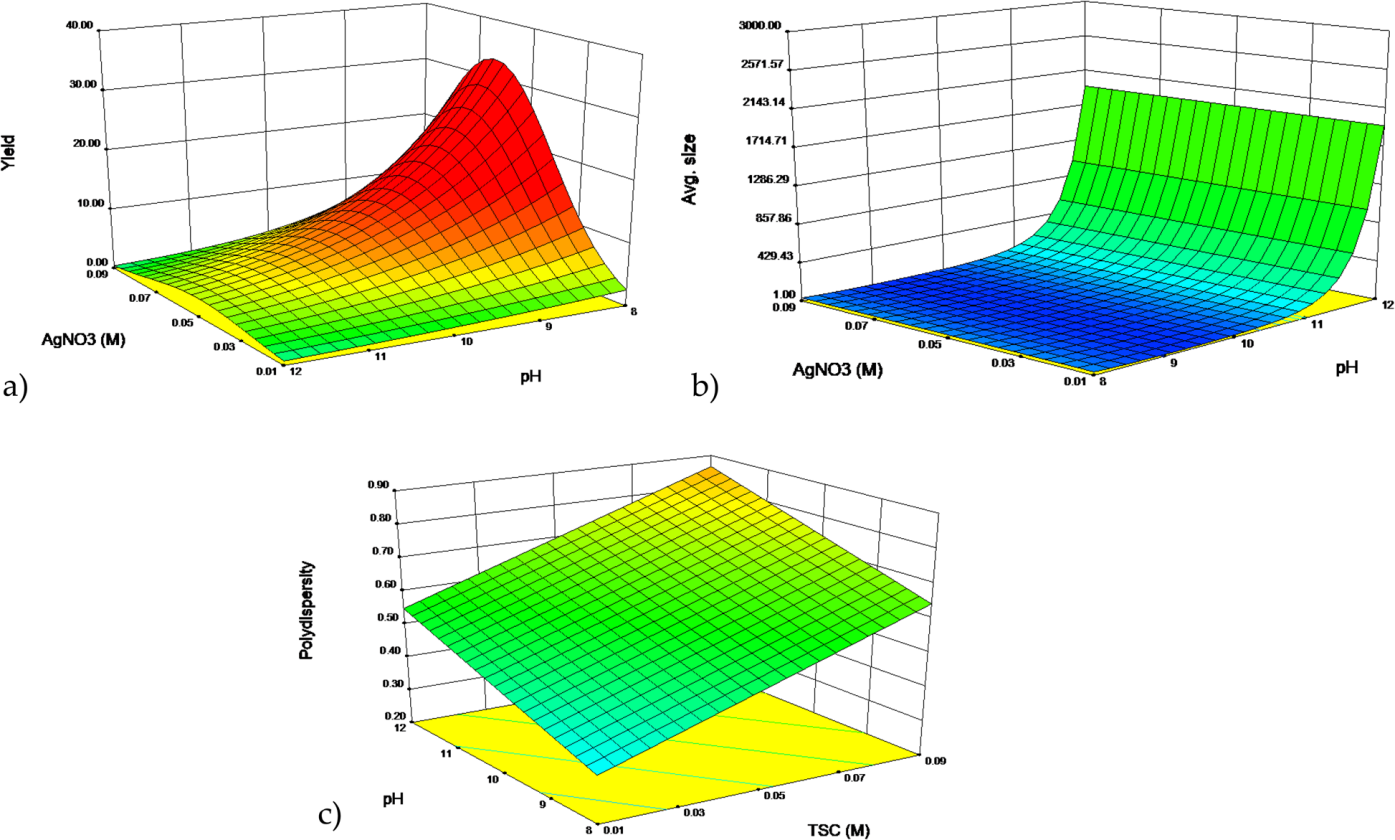
3D interaction plot of AgNPs. **a** interaction of pH and *AgNO*_3_ on the production performance of AgNPs; **b** interaction of concentration pH and *AgNO*_3_ on the average size of AgNPs; **c** interaction of pH and concentration of *AgNO*_3_ on the polydispersity of the size of AgNPs

Additionally, Fig. 5 shows the response surfaces with greater significance for the yield, size and polydispersity of the size of AgNPs using the interactions of three variables. Figure 5a shows that when increasing pH and concentration of *AgNO*_3_ is between 0.05 and 0.07 M, the yield in the production of AgNPs increases, hence, this image suggests that there are optimal conditions related to pH and *AgNO*_3_ concentration to increase the production of AgNPs. In addition, the size effect of AgNPs from pH is observed in Fig. 5b in which, the size of the particles increases when pH increases although the concentration of *AgNO*_3_ varies. Finally, Fig. 5c shows the effect of the concentration of *AgNO*_3_ and pH in the polydispersity of the AgNPs size, showing that the polydispersity decreases by reducing pH.

Based on the statistical analysis obtained, Table 3 shows the limits and importance of each parameter as well as the optimal values of the parameters with the predicted responses for each response variable.

**Table 3:** Restrictions and optimal conditions predicted from the model obtained

Parameter	Lower limit	Upper limit	Importance	Optimal value	Predicted value	Experimental results	Relative error
TSC	0.01	0.09	3	0.01	—	—	—
*AgNO*_3_	0.01	0.09	3	0.06	—	—	—
*NaBH*_4_	0.01	0.09	3	0.01	—	—	—
pH	8	12	3	8	—	—	—
Response variable							
Yield	0	15.00	5	—	2.61	0.97 ±0.00	1.64
Size	13.65	37850	3	—	86.67	0.94 ±0.05	76.73
PSGI	15.17	37850	4	—	30.99	62.63 ±2.79	-31.64
Polydipersity	0.112	1	5	—	0.33	0.83 ±0.00	-0.50

### Characterization of AgNPs

#### Physico-chemical characterization of AgNPs

**Fig. 6:**
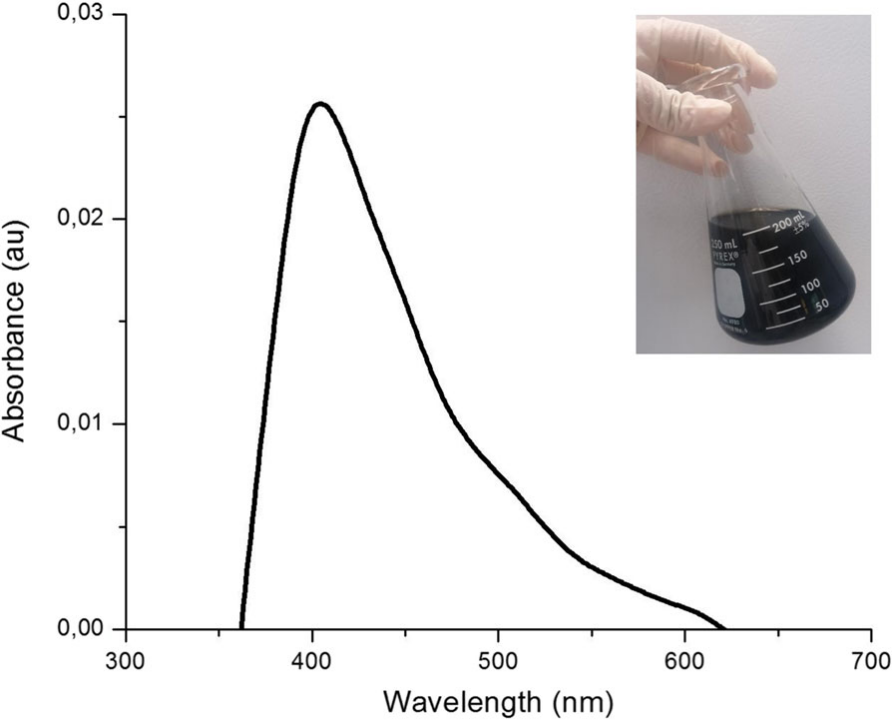
UV-Vis spectroscopy of optimized AgNPs [1%] (v/v)

The formation of optimized AgNPs was confirmed through the UV-Vis absorption spectrum (Fig. 6), wavelengths were observed at 400 nm and no absorption peaks were observed indicating the presence of residues of the synthesis process in the range of evaluated length. Table 4 shows the results of the characterization by AAS and DLS of AgNPs.

**Table 4:** Physico-chemical characterization of AgNPs

Characteristic of AgNPs	Values to Opt-AgNPs
Concentration by AAS (*μ*g/mL)	77.8
Average hydrodynamic size by DLS (nm)	9.94
Zeta potential by DLS (mV)	-3.96

**Fig. 7:**
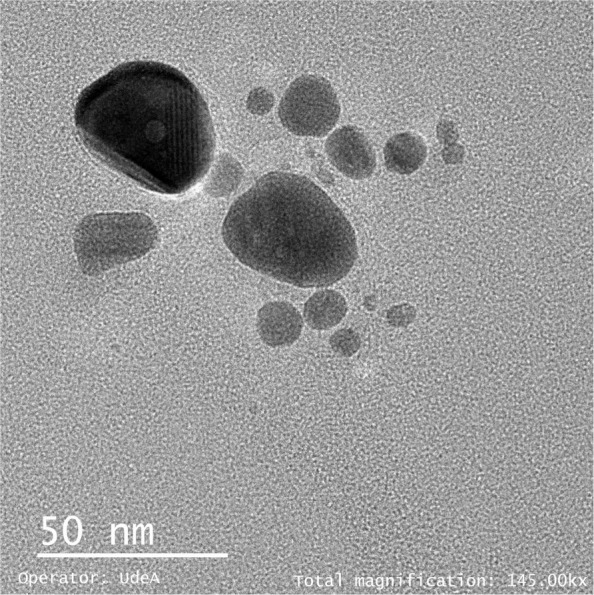
Micrograph of AgNPs taken with TEM at magnification of 50 nm

In addition, Fig. 7 shows TEM micrographs for Opt- AgNPs. The AgNPs were observed with a heterogeneous distribution with variable spherical trend morphology.

#### Evaluation of the antimicrobial effect of AgNPs

**Fig. 8:**
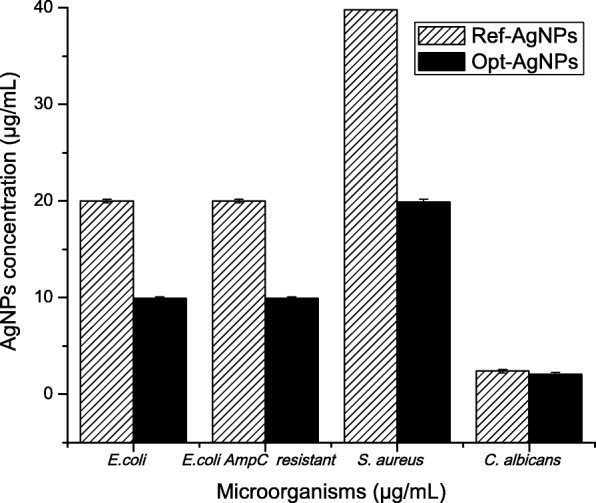
Comparison between the MBC and MFC of the reference AgNPs and the optimized AgNPs

Table 5 shows the bactericidal and fungicidal effect of AgNPs. It was found better antimicrobial activity in optimized nanoparticles than in reference. Also, its antifungical effect against *Candida albicans* was greater with the AgNPs than the control of fluconazole, which was evaluated at a concentration of 10 *μ*g/mL. Growth and sterility controls were appropriate for all essays. Also, Fig. 8 shows these results as a comparison between the two AgNPs using a bar chart.

**Table 5:** Antimicrobial activity of AgNPs at 24h incubation

Microorganisms	Ref-AgNPs	Opt-AgNPs
	MBC (*μ*g/mL)	
*Escherichia coli* ATCC 25922	20	9.94
*Escherichia coli* AmpC resistant	20	9.94
*Staphylococcus aureus* ATCC 25923	>39.78	19.89
	MFC (*μ*g/mL)	
*Candida albicans* ATCC 14053	2.40	2.08

MBC: Minimum Bactericidal Concentration; MFC: Minimum Fungicidal Concentration

#### Evaluation of the cytotoxic effect of optimized AgNPs

**Fig. 9:**
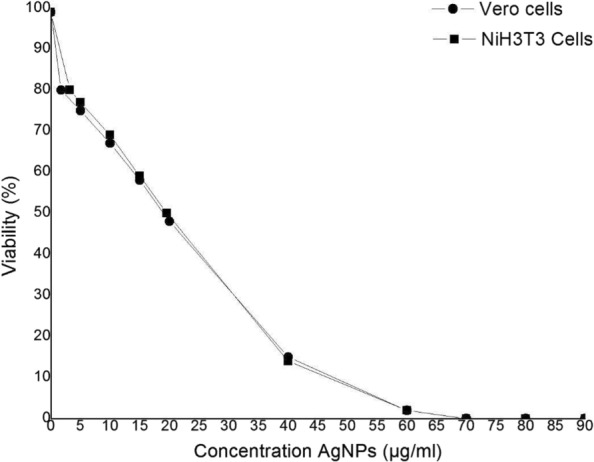
Cell viability in Vero and NiH3T3 cells exposed to AgNPs

Figure 9 shows the viability percentage of Vero and NiH3T3 cells according to the different concentrations of AgNPs. It was found that the viability of both cells decreased as the concentration of AgNPs increased. Besides, no significant differences were found between the cell lines evaluated.

Furthermore, *LC*_20_ and *LC*_50_ of AgNPs was found on 1.74 *μ*g/mL and 19.11 *μ*g/mL to Vero cells, respectively. *LC*_20_ and *LC*_50_ of AgNPs was found on 3.21 *μ*g/mL and 19.60 *μ*g/mL to NiH3T3 cells, respectively.

## Discussion

The novelty in this article is the use of FCCCD, using the RSM, to optimize the physico-chemical properties and antimicrobial activity of silver nanoparticles presented as Ref-AgNPs. This work shows that it is possible to improve the antimicrobial activity of the optimized AgNPs by comparing with the initial AgNPs, using the same method, but making changes in four parameters: *AgNO*_3_ concentration, TSC concentration, *NaBH*_4_ concentration and pH of the solution.

For this purpose, an optimization process of AgNPs was carried out, starting from an initial chemical reduction synthesis process. Optimized AgNPs were obtained, and their physico-chemical properties, antimicrobial activity, and cytotoxic effect were evaluated.

The FCCCD results showed the formulation that delivered the highest yield in the production of AgNPs was the synthesis that used the highest concentration of the three reagents and a final pH adjustment to 8.This may be related to the fact that, the higher the concentration of the metal precursor and the reducing agent, the greater the possibility of obtaining AgNPs, due to the availability of the substrate and the ability of *NaBH*_4_ to release electrons to the oxidizing agent and reduce the silver in nanoparticles [17].

In addition, it was found that the synthesis with the lowest average nanoparticle size, the peak of greatest intensity of the lowest size and the lowest polydispersity were prepared with *AgNO*_3_ [0.01 M] and pH 8. The explanation to this is related to the way the synthesis process was performed with the lowest concentration of the evaluated precursor agent, which had the opportunity to be more exposed to the reducing agent, forming electric layers around the nanoparticles that inhibit aggregation and reduce the size [17]. Furthermore, it has been found that the size of nanoparticles depends on the speed of nucleation and growth process, which can be controlled by parameters such as pH [29]. Ondari et al. found that lower synthesis pH generated smaller nanoparticle sizes [29].

Likewise, some synthesis processes were carried out with pH 12, in which no area under the curve of the UV-Vis spectrum was found between the lengths of 350 nm and 420 nm and in which larger sizes of AgNPs and polydispersity of size were recorded. It is possible that the parameters established for the concentration of the three reagents and the pH did not allow the formation of AgNPs with sizes between 5 and 50 nm [30] and large silver clusters have been formed that could not be adequately measured by the equipment of size measurement. Similary, it has been found that synthesis formulations with pH 12 can affect the size of AgNPs. Tagad et al. [31] synthesized AgNPs and evaluated the effect of pH on the size of the nanoparticles in the different reactions at pH. The authors found an agglomeration of AgNPs in the synthesis with the highest pH. This could explain the results obtained in our study, where an extreme alkaline pH can generate low stability.

Futhermore, when validating the models and observing the normal residual graphs for each model, some distances was found between the predicted values and the real values, due to the experimental conditions related to the synthesis processes. However, these follow a normal distribution, endorsing the models [32].

In this study, the DOE developed showed pH, *AgNO*_3_ and pH interactions, *TSC*^2^ and *AgNO*_3_ are significant factors for the yield in the production of AgNPs by this method. Likewise, pH, *AgNO*_3_ and protective agent influenced the size of the nanoparticles obtained, as has been reported in other studies [14, 29]. Considering that the nanoparticles are made up *AgNO*_3_, the concentration of this reagent is determinant for the production yield of AgNPs. Moreover, the concentration of TSC has greater effect on the size of AgNPs due to its role of preventing the aggregation of the nanoparticles. Subsequently, it was found that *NaBH*_4_ has a great effect on the polydispersity of the AgNPs size, since *NaBH*_4_ is a strong reducer, which allows that the reaction rate in the nucleation stage of the synthesis to be greater [33], whereby, the silver ions have less time to generate clusters of variable sizes.

On the other hand, it was possible to verify that the AgNPs obtained with the optimization of the chemical synthesis corresponded to silver when evaluating LSPR by UV-Vis. The observed bands showed a widening that suggests a distribution of different sizes of nanoparticles. Also, adequate yield was found in the production of AgNPs evaluated by AAS and a slightly negative Zeta Potential since *NaBH*_4_ was used as a reducing agent and the nanoparticles formed absorbed the nitrate and borate ions that are slightly negative. For instance, with TEM, it was possible to observe individual nanoparticles of the synthesis carried out. These results are related to those obtained by other authors [1, 21, 22] who have shown that chemical methods allow obtaining small nanoparticles with a spherical tendency.

It has been found that the size, oxidation, and release capacity of AgNPs are factors that are associated with their antimicrobial activity [1, 34, 35]. The micro and macrodilutions methodology used to evaluate the antimicrobial capacity of the synthesized AgNPs allowed us to know their MBC and MFC against all the microorganisms evaluated. The fact that changing the parameters of the formulation can decrease the lowest concentration of the nanoparticles required to kill some bacterias and fungi, reveals the importance of the physico-chemical properties in its antimicrobial capacity.

The results obtained indicate that the nanoparticle solution was not monodispersed, since the particles were not obtained of a uniform size. Several factors can generate these results, among which are the preparation and reaction conditions of the synthesis method used. It has been found that the rate of incorporation of the reagents, the agitation of the mixture, and the reaction rate determine the size distribution of AgNPs obtained [36]. This paper presents a TEM image to demonstrate the formation of Opt-AgNPs. Nanoparticles of different sizes are observed; however, this is an image from a portion of the sample. The polydispersity of the nanoparticle size was calculated by the DLS technique and described in Table 3

As in our study, other authors have found antimicrobial activity of AgNPs, synthesized by chemical reduction, against *Staphylococcus aureus*, *Escherichia coli*, and *Candida albicans* [1, 21, 22, 35]. Three toxicity mechanisms of AgNPs against microorganism have been established [4, 5, 7, 8].

All these toxicity mechanisms of AgNPs begin with the adhesion and permeability of the membrane of the microorganisms. However, Gram-positive bacteria have a greater thickness of the cell wall through the peptidoglycan layer (30 to 100 nm thick) than Gram-negative bacteria [37]. This could explain the difference in the MBC between *Staphylococcus aureus* and *Escherichia coli* [37]. Another explanation for this phenomenon can be related to the presence of lipoteichoic acid in Gram-positive bacteria, which protect these microorganisms against external agents [37]. Nevertheless, it was found greater sensitivity of *Candida albicans* to AgNPs comparing to bacteria’s sensitivy, which can be attributed to the large number of functional groups present on the surface of bacteria with respect to that of fungi [38].

In particular, it was found that AgNPs obtained in this study achieved a MBC against *Staphylococcus aureus*, unlike AgNPs before the optimization of the synthesis parameters. Also, this study found that the optimized nanoparticles possessed a higher toxicity against *Escherichia coli*, *Escherichia coli* AmpC resistant and *Candida albicans* compared to the reference AgNPs. These results can be attributed to differences in the concentrations of TSC, *AgNO*_3_ and *NaBH*_4_ between both formulations, where the protective and reducing agents of the optimized synthesis were lower than the initial while the metallic precursor increased.

in addition, other authors have used some of the same reagents to synthesize AgNPs used in this work. However, differences have been found in the concentrations used and in the antimicrobial effects of nanoparticles.

By comparison, Raji et al. [1] synthesized AgNPs using *AgNO*_3_ [0.1M] and *NaBH*_4_ as a reducing agent. They found a MIC for *Escherichia coli* and *Staphylococcus aureus* of 1.39 *μ*g/mL and 5.5 mg/mL against *Candida albicans* after 24 and 48 h of incubation. Thus, the nanoparticles optimized in our study required 8.55 *μ*g/mL and 18.5 *μ*g/mL more than Raji’s to achieve inhibition of *Escherichia coli* and *Staphylococcus aureus*. However, 5.5 ×10^−3^*μ*g/mL less of our optimized AgNPs is required to inhibit the growth of *Candida albicans* compared to Raji, which is a big difference. This could be related to the difference in reagent concentration. It should be noted that some authors do not specify the type of strain used in each microorganism. Additionally, these authors did not use a design of experiments to improve the antimicrobial activity of their nanoparticles, optimizing some parameters of their synthesis process like this work.

Furthermore, previous studies have linked the antimicrobial activity of spherical AgNPs with its size, and other characteristics such as oxidation capacity and the release of silver ions [1, 34, 35]. Also, because the antimicrobial effect of AgNPs on bacteria and fungi is affected by the interaction of these nanoparticles with microorganisms, it has been claimed that smaller nanoparticles may have higher antimicrobial activity compared to larger [1]. This, because smaller nanoparticles have a larger surface area available to interact with microorganisms and release more ions [19, 19]. However, larger AgNPs may have less toxic effects on human cells than those of small sizes [19]. Jeong et al. [19] prepared two different sizes of AgNPs (10 and 100 nm in average diameters) with similar chemical composition and using an *AgNO*_3_ reduction method like the one of the present work. The authors found that smaller particles showed a higher cytotoxic effect, at the same concentration, compared to larger particles.

The evaluation of the cytotoxic effect of optimized AgNPs was carried out through cellular viability by MTT assay on cells NiH3T3 and Vero. This type of testing is necessary to determine the cytotoxicity of any product that is intended for use in humans [39]. It was found that cell viability and cytotoxicity dependence of AgNPs concentration [40, 41]. This occurs because, at a higher concentration of AgNPs, cells are more prone to damage in the cell membrane, which produces permeability in the mitochondrial membrane and greater exposure to Ag ions [42]. For this reason, at concentrations less than 13.88 *μ*g/mL and 14.66 *μ*g/mL AgNPs, the viability of cells was above 60% for NiH3T3 and Vero cells. However, concentration of 20 *μ*g/mL AgNPs reduced the viability to 50%.

It was found that *LC*_20_ and *LC*_50_ of AgNPs were lower for Vero than NiH3T3 cells. This is probably because of some kind of cells or cellular lines can be more sensitive than other types of cells to nanoparticles [41, 43]. In this case, Vero cells were more sensitive than fibroblast cells (NiH3T3). Other studies have also studied the cytotoxic effect of AgNPs [44, 45]. Accordingly, it is possible to employ our AgNPs at concentrations less than 10 *μ*g/mL to achieve bactericidal activity against *Escherichia coli*, *Escherichia coli* AmpC resistant, and *Candida albicans* and ensure a viability of 70% for Vero and NiH3T3 cells. However, it is not advisable to use these nanoparticles with concentrations greater than 20 *μ*g/mL to eradicate *Staphylococcus aureus*, since the viability of Vero and NiH3T3 cells would be significantly affected.

To inhibit the growth of *Staphylococcus aureus*, a minimum inhibitory concentration could be used, which may be less than the MBC. Thomas et al. evaluated the antimicrobial activity of AgNPs against *Staphylococcus aureus* and found an MBC and MIC of 62.5 *μ*g/mL and 1.95 *μ*g/mL, respectively [46]. Similarly, Du et al. synthesized AgNPs and found an MBC of 100 *μ*g/mL and a MIC of 50 *μ*g/mL against the same microorganism [47]. These studies suggest that MBC may be higher than the MIC of AgNPs for the same bacteria.

Lastly, it is not clear what is the ideal size distribution of AgNPs that guarantees low antimicrobial activity and avoids the toxic effects of AgNPs for health as much as possible. Nevertheless, the size distribution of the AgNPs optimized in this study could have generated a lower cytotoxic effect on Vero and NiH3T3 cells. Likewise, the smallest nanoparticles of optimized AgNPs size distribution and the ability to release silver ions from the larger nanoparticles could generate the antimicrobial effect against the microorganisms evaluated.

## Conclusion

In this study, a face centered central composite design (FCCCD) through response surface methodology (RSM) was applied to optimize the chemical reduction synthesis of AgNPs. The objective was increasing the antimicrobial capacity of a reference AgNPs through the optimization of some synthesis factors. The experimental results confirmed that all the factors studied were significant for the variable responses. AgNPs optimized with an average size of 9.94 nm and spherical and hemispherical shapes were obtained. Higher antimicrobial activity was found in optimized AgNPs than in reference AgNPs against *Escherichia coli*, *Escherichia coli* AmpC resistant, and *Candida albicans* and was necessary 9.94, 9.94 and 2.08 *μ*g/mL of AgNPs, respectively, to eliminate them. Further, it was achieved bactericidal effect against *Staphylococcus* at a concentration of 19.89 *μ*g/mL AgNPs. It was also found that optimized AgNPs show no significant cytotoxicity against Vero and NiH3T3 cells and allowed a minimum viability of 70% at concentrations less than 10 *μ*g/mL AgNPs.

This work is the first study that optimizes the process of obtaining AgNPs with the design of experiments from the synthesis method presented in this work, and in which a better antimicrobial effect was achieved compared to the reference AgNPs. This work shows that it is possible to improve the antimicrobial activity of AgNPs obtained by a specific method, altering some parameters, without changing that methodology. This could be applied in those cases in which, for reasons of availability of other methods or lack of resources, it is not possible to change the methodology of synthesis chosen.

Future works may also consider using different parameters (for example, stirring time, mixing RPM, and reaction temperature) for the optimization of AgNPs that allow reducing minimum bactericidal concentration (MBC) and minimum fungicidal concentration (MFC) against microorganisms and avoid reducing cell viability.

## Data Availability

All data generated or analysed during this study are included in this published article.

## Abbreviations

AAS: Atomic absorption spectroscopy
AgNPs: Silver nanoparticles
ANOVA: Analysis of variance
ATCC: American type culture collection
ATP: Adenosine triphosphate
BHI: Brain heart infusion
CFU: Colony Forming Units
DLS: Dynamic light scattering
DMEM: Dulbecco’s modified eagle Medium
DMSO: Dimethylsulfoxide
DNA: Deoxyribonucleic acid
DOE: Design of experiments
*L**C*_20_: Lethal concentration 20
*L**C*_50_: Lethal concentration 50
MBC: Minimum bactericidal concentration
MFC: Minimum fungicidal concentration
MIC: Minimum inhibitory concentration
MTT: 3-[4,5-dimethylthiazol-2-yl]-diphenyl tetrazolium bromide
OECD: Economic Cooperation and Development
PSGI: Peak with the greatest intensity in the size distribution
SFB: Fetal bovine serum
SPR: Surface plasmon resonance
RSM: Response Surface Methodology
TEM: Transmission electron microscopy
TSC: Sodium citrate
UV-Vis: Ultraviolet- visible spectroscopy

## References

[1] Raji V, Chakraborty M, Parikh PA (2012). Synthesis of Starch-Stabilized Silver Nanoparticles and Their Antimicrobial Activity. Part Sci Technol.

[2] Karwowska E (2016). Antibacterial potential of nanocomposite-based materials- a short review. Nanotechnol Rev.

[3] Davoodbasha M, Kim S-C, Lee S-Y, Kim J-W (2016). The facile synthesis of chitosan-based silver nano-biocomposites via a solution plasma process and their potential antimicrobial efficacy. Arch Biochem Biophys.

[4] Rai M, Yadav A, Gade A (2009). Silver nanoparticles as a new generation of antimicrobials. Biotechnol Adv.

[5] Dos Santos CA, Seckler MM, Ingle AP, Gupta I, Galdiero S, Galdiero M, Gade A, Rai M (2014). Silver nanoparticles: Therapeutical uses, toxicity, and safety issues. J Pharm Sci.

[6] Eremenko AM, Petrik IS, Smirnova NP, Rudenko AV, Marikvas YS (2016). Antibacterial and Antimycotic Activity of Cotton Fabrics, Impregnated with Silver and Binary Silver/Copper Nanoparticles. Nanoscale Res Lett.

[7] Wong KKY, Liu X (2010). Silver nanoparticles - the real "silver bullet" in clinical medicine?,. Med Chem Commun.

[8] Durán N, Durán M, de Jesus MB, Seabra AB, Fávaro WJ, Nakazato G (2016). Silver nanoparticles: A new view on mechanistic aspects on antimicrobial activity. Nanomed Nanotechnol Biol Med.

[9] Liu B, Han G (2011). Shell thickness-dependent raman enhancement for rapid identification and detection of pesticide residues at fruit peels. Anal Chem.

[10] Alessio P, Aoki PHB, Furini LN, Aliaga AE, Constantino CJL. Spectroscopic Techniques for Characterization of Nanomaterials In: Da Róz AL, Ferreira M, Leite FdL, Osvaldo N OJ, editors. Nanocharacterization Techniques. 1st edn. Elsevier Inc.: 2017. p. 65–98. Chap. 3. 10.1016/B978-0-323-49778-7/00003-5.

[11] Filipe V, Hawe A, Jiskoot W (2010). Critical evaluation of nanoparticle tracking analysis (NTA) by nanosight for the measurement of nanoparticles and protein aggregates. Pharm Res.

[12] Brar K S, Vierma M (2011). Measurement of nanoparticles by light-scattering techniques. TrAC Trends Anal Chem.

[13] Leng Y (2013). Materials Characterization. Introduction to Microscopic and Spectroscopic Methods.

[14] Hasnain MS, Javed MN, Alam MS, Rishishwar P, Rishishwar S, Ali S, Nayak AK, Beg S (2019). Purple heart plant leaves extract-mediated silver nanoparticle synthesis: Optimization by Box-Behnken design. Mater Sci Eng C.

[15] Núñez RN, Veglia AV, Pacioni NL (2018). Improving reproducibility between batches of silver nanoparticles using an experimental design approach. Microchem J.

[16] Ajitha B, Reddy YA, Reddy P (2015). Enhanced antimicrobial activity of silver nanoparticles with controlled particle size by pH variation. Powder Technol.

[17] Brown AN, Smith K, Samuels TA, Lu J, Obare SO, Scott ME (2012). Nanoparticles functionalized with ampicillin destroy multiple-antibiotic-resistant isolates of Pseudomonas aeruginosa and Enterobacter aerogenes and methicillin-resistant Staphylococcus aureus. Appl Environ Microbiol.

[18] Chueh PJ, Liang RY, Lee YH, Zeng ZM, Chuang SM (2014). Differential cytotoxic effects of gold nanoparticles in different mammalian cell lines. J Hazard Mater.

[19] Yoon J, Dong Woo L, Choi J (2014). Assessment of Size-Dependent Antimicrobial and Cytotoxic Properties of Silver Nanoparticles. Adv Mater Sci Eng.

[20] Zapata-Giraldo J, Mena P, Cuesta D, Galeano B, Mejía M, Botero LE, Ortiz I, Escobar N, Hoyos L (2016). Characterization of silver nanoparticles for potential use as antimicrobial agent. VII Congreso Latinoamericano de Ingeniería Biomédica.

[21] Monteiro DR, Gorup LF, Silva S, Negri M, de Camargo ER, Oliveira R, Barbosa DB, Henriques M (2011). Silver colloidal nanoparticles: antifungal effect against adhered cells and biofilms of Candida albicans and Candida glabrata. Biofouling.

[22] Panáček A, Kolář M, Večeřová R, Prucek R, Soukupová J, Kryštof V, Hamal P, Zbořil R, Kvítek L (2009). Antifungal activity of silver nanoparticles against Candida spp,. Biomaterials.

[23] Mahl D, Diendorf J, Meyer-Zaika W, Epple M (2011). Possibilities and limitations of different analytical methods for the size determination of a bimodal dispersion of metallic nanoparticles. Colloids Surf A Physicochem Eng Asp.

[24] Kruk T, Szczepanowicz K, Stefańska J, Socha RP, Warszyński P (2015). Synthesis and antimicrobial activity of monodisperse copper nanoparticles. Colloids Surf B Biointerfaces.

[25] Kumar SV, Bafana AP, Pawar P, Faltane M, Rahman A, Dahoumane SA, Kucknoor A, Jeffryes CS (2019). Optimized production of antibacterial copper oxide nanoparticles in a microwave-assisted synthesis reaction using response surface methodology. Colloids Surf A Physicochem Eng Asp.

[26] Krishnan R, Vijay A, Vasaviah SK. The MIC and MBC of Silver Nanoparticles against Enterococcus faecalis - A Facultative Anaerobe. J Nanomedicine Nanotechnol. 2015; 06(03). 10.4172/2157-7439.1000285.

[27] Mosmann T (1983). Rapid Colorimetric assay for cellular growth and survival: application to proliferation and cytotoxicity assay. J Immunol Methods.

[28] Fertig J (1948). Probit Analysis: A Statistical Treatment of the Sigmoid Response Curve. D. J. Finney. Q Rev Biol.

[29] Ondari Nyakundi E, Padmanabhan MN (2015). Green chemistry focus on optimization of silver nanoparticles using response surface methodology (RSM) and mosquitocidal activity: anopheles stephensi (diptera: culicidae). Spectrochim Acta Part A Mol Biomol Spectrosc.

[30] Chowdhury Silvia, Yusof Faridah, Faruck Mohammad Omer, Sulaiman Nadzril (2016). Process Optimization of Silver Nanoparticle Synthesis Using Response Surface Methodology. Procedia Engineering.

[31] Park S, Aiyer R, Sabharwal S, Dugasani SR, Kulkarni A, Tagad CK (2013). Green synthesis of silver nanoparticles and their application for the development of optical fiber based hydrogen peroxide sensor. Sensors Actuators B Chem.

[32] Draper NR, Smith H. Applied Regression Analysis, 3rd edn: Wiley-Interscience; 2014, p. 736. 10.1002/9781118625590.

[33] Sharma VK, Yngard RA, Lin Y (2009). Silver nanoparticles: Green synthesis and their antimicrobial activities. Adv Colloid Interface Sci.

[34] GF P, AS P, NP K, SA K, AI E, TG E, TV F, LM. S (2014). Green synthesis of water-soluble nontoxic polymeric nanocomposites containing silver nanoparticles. Int J Nanomedicine.

[35] Cakić M, Glišić S, Nikolić G, Nikolić GM, Cakić K, Cvetinov M (2016). Synthesis, characterization and antimicrobial activity of dextran sulphate stabilized silver nanoparticles. J Mol Struct.

[36] Viudez AJ (2011). Síntesis, caracterización y ensamblaje de nanopartículas de oro protegidas por monocapas moleculares. Ph.d. thesis, Universidad de Córdoba.

[37] Mohanbaba Subhashini, Gurunathan Sangiliyandi (2016). Differential biological activities of silver nanoparticles against Gram-negative and Gram-positive bacteria. Nanobiomaterials in Antimicrobial Therapy.

[38] Biao L, Tan S, Wang Y, Guo X, Fu Y, Xu F, Zu Y, Liu Z (2017). Synthesis, characterization and antibacterial study on the chitosan-functionalized Ag nanoparticles. Mater Sci Eng C.

[39] Packirisamy G, Gogoi S, Chattopadhyay A, Ghosh S (2008). Implications of silver nanoparticle induced cell apoptosis for in vitro gene therapy. Nanotechnology.

[40] Brennan SA, Ní Fhoghlú C, Devitt BM, O’Mahony FJ, Brabazon D, Walsh A (2015). Silver nanoparticles and their orthopaedic applications. Bone Joint J.

[41] Ahamed M, AlSalhi MS, Siddiqui MKJ (2010). Silver nanoparticle applications and human health. Clin Chimica Acta.

[42] Dos Santos CA, Seckler MM, Ingle AP, Gupta I, Galdiero S, Galdiero M, Gade A, Rai M (2014). Silver nanoparticles: Therapeutical uses, toxicity, and safety issues. J Pharm Sci.

[43] Nuñez-Anita RE, Acosta-Torres LS, Vilar-Pineda J, Martínez-Espinosa JC, De la fuente-Hernández J, Castaño VM (2014). Toxicology of antimicrobial nanoparticles for prosthetic devices. Int J Nanomedicine.

[44] Kasithevar M, Saravanan M, Prakash P, Kumar H, Ovais M, Barabadi H, Shinwari ZK (2017). Green synthesis of silver nanoparticles using Alysicarpus monilifer leaf extract and its antibacterial activity against MRSA and CoNS isolates in HIV patients. J Interdiscip Nanomedicine.

[45] Barbalinardo M, Caicci F, Cavallini M, Gentili D (2018). Protein Corona Mediated Uptake and Cytotoxicity of Silver Nanoparticles in Mouse Embryonic Fibroblast. Small.

[46] Thomas R, Mathew S, Nayana AR, Mathews J, Radhakrishnan EK (2017). Microbially and phytofabricated AgNPs with different mode of bactericidal action were identified to have comparable potential for surface fabrication of central venous catheters to combat Staphylococcus aureus biofilm. J Photochem Photobiol B Biol.

[47] Du J, Hu Z, Yu Z, Li H, Pan J, Zhao D, Bai Y (2019). Antibacterial activity of a novel Forsythia suspensa fruit mediated green silver nanoparticles against food-borne pathogens and mechanisms investigation. Mater Sci Eng C.

